# The Impact of DNA Extraction Methods on Stool Bacterial and Fungal Microbiota Community Recovery

**DOI:** 10.3389/fmicb.2019.00821

**Published:** 2019-04-17

**Authors:** Kristýna Fiedorová, Matěj Radvanský, Eva Němcová, Hana Grombiříková, Juraj Bosák, Michaela Černochová, Matej Lexa, David Šmajs, Tomáš Freiberger

**Affiliations:** ^1^Centre for Cardiovascular Surgery and Transplantation, Brno, Czechia; ^2^Central European Institute of Technology, Masaryk University, Brno, Czechia; ^3^Department of Clinical Immunology and Allergology, Faculty of Medicine, Masaryk University, Brno, Czechia; ^4^Faculty of Informatics, Masaryk University, Brno, Czechia; ^5^Department of Biology, Faculty of Medicine, Masaryk University, Brno, Czechia

**Keywords:** gut microbiome, gut microbiota, gut mycobiome, gut mycobiota, fungal microbiota, DNA extraction method, 16S rDNA, ITS rDNA

## Abstract

Our understanding of human gut microbiota in health and disease depends on accurate and reproducible microbial data acquisition. The critical step in this process is to apply an appropriate methodology to extract microbial DNA, since biases introduced during the DNA extraction process may result in inaccurate microbial representation. In this study, we attempted to find a DNA extraction protocol which could be effectively used to analyze both the bacterial and fungal community. We evaluated the effect of five DNA extraction methods (QIAamp DNA Stool Mini Kit, PureLink^TM^ Microbiome DNA Purification Kit, ZR Fecal DNA MiniPrep^TM^ Kit, NucleoSpin^®^ DNA Stool Kit, and IHMS protocol Q) on bacterial and fungal gut microbiome recovery using (i) a defined system of germ-free mice feces spiked with bacterial or fungal strains, and (ii) non-spiked human feces. In our experimental setup, we confirmed that the examined methods significantly differed in efficiency and quality, which affected the identified stool microbiome composition. In addition, our results indicated that fungal DNA extraction might be prone to be affected by reagent/kit contamination, and thus an appropriate blank control should be included in mycobiome research. Overall, standardized IHMS protocol Q, recommended by the International Human Microbiome Consortium, performed the best when considering all the parameters analyzed, and thus could be applied not only in bacterial, but also in fungal microbiome research.

## Introduction

The human gut microbiome has been a subject of study for more than a century (Mattill and Hawk, 1911; Rogers et al., 1918), but with the recent advent of new culture-independent technologies (i.e., massive parallel sequencing), it has attracted enormous attention. Numerous researchers have described its association with a number of health benefits related to pathogen protection, nutrition, metabolism, and immune functions (Clemente et al., 2012; Lozupone et al., 2012; Pascale et al., 2018). Simultaneously, unfavorably altering the composition and function of the microbiota, known as dysbiosis, alters the host–microbiota interaction and the host’s immune system. Gut microbiota dysbiosis was shown to be associated with human diseases including inflammatory bowel disease (IBD), colorectal cancer, obesity, diabetes, and also non-intestinal conditions including atopic dermatitis, asthma, cardiovascular diseases, behavior disorders and many others (Turnbaugh et al., 2006; Maloy and Powrie, 2011; Qin et al., 2012; Louis et al., 2014; Zheng et al., 2016; Cani, 2017; Salem et al., 2018).

The majority of this research was primarily focused on bacteria, but the role of fungal communities (known as mycobiota) in human health has recently emerged. Mycobiota represents only 0.1% of total gut microbiota (Qin et al., 2010), but they are also important for gut homeostasis (Wheeler et al., 2016), since fungi affect bacterial microbiota and host physiology (Underhill and Iliev, 2014; Lamprinaki et al., 2017). Moreover, fungal dysbiosis has recently been associated with IBD (Sokol et al., 2017; Miyoshi et al., 2018) or recurrences of *Clostridium difficile* infection after fecal microbiota transplantation (Zuo et al., 2018).

During the intensive worldwide study of gut microbiomes, the use of different methodologies to prepare samples resulted in numerous microbiome studies with contradictory results. Therefore, one of the presumptions for reproducible and comparable microbiome research is the use of appropriate methods to collect specimens, and extract and store DNA (McOrist et al., 2002; Salonen et al., 2010; Wesolowska-Andersen et al., 2014; Rintala et al., 2017; Lim et al., 2018; Velásquez-Mejía et al., 2018). In an effort to standardize current methodology, the International Human Microbiota Consortium (IHMC) performed the International Human Microbiota Standards (IHMS) project. There, twenty-one DNA extraction protocols from human fecal samples widely used across laboratories were compared. Protocol Q (Doré et al., 2015), formerly known as the repeated bead beating column method (RBBC) (Yu and Morrison, 2004), provided the most appropriate results according to quality, transferability and reproducibility, and thus was proposed as the standard operating protocol (Costea et al., 2017) with the ambition of serving as the benchmark for newly developed protocols. However, the IHMS recommended protocol Q was designed exclusively for bacterial microbiota analyses and its impact on fungal communities was not evaluated.

Therefore, gut mycobiome research is facing the same lack of the standardization as bacterial microbiome research in the past. To our knowledge, only two studies have attempted to evaluate the effect of different extraction protocols on the outcome of human gut mycobiome research (Huseyin et al., 2017; Angebault et al., 2018). In these two studies, the impact of three protocols (protocol Q vs. QIAamp Fast DNA Stool Mini Kit with Bead beating and protocol Q vs. MoBio PowerLyzer PowerSoil^®^ DNA Isolation kit) on fungal or combined bacterial and fungal communities were investigated. Despite their effort, mycobiome research is still extremely underexplored and more studies focused on assessing and validating methodology are needed. Most importantly, a protocol capable of accurately extracting DNA from both microbial communities needs to be established.

In this study, we aimed to expand on current knowledge of how DNA extraction methods affect both bacterial and fungal gut community recovery. Five DNA extraction methods (QIAamp DNA Stool Mini Kit, PureLink^TM^ Microbiome DNA Purification Kit, ZR Fecal DNA MiniPrep^TM^ Kit, NucleoSpin^®^ DNA Stool Kit, and IHMS protocol Q) were evaluated using (i) a defined system of germ-free mice feces spiked with bacterial or fungal strains, and (ii) non-spiked human feces; in order to compare the efficiency of microbial DNA recovery and microbial composition profiles. The presence of kit/reagent fungal contamination was also addressed.

## Materials and Methods

### Study Design

**FIGURE 1:**
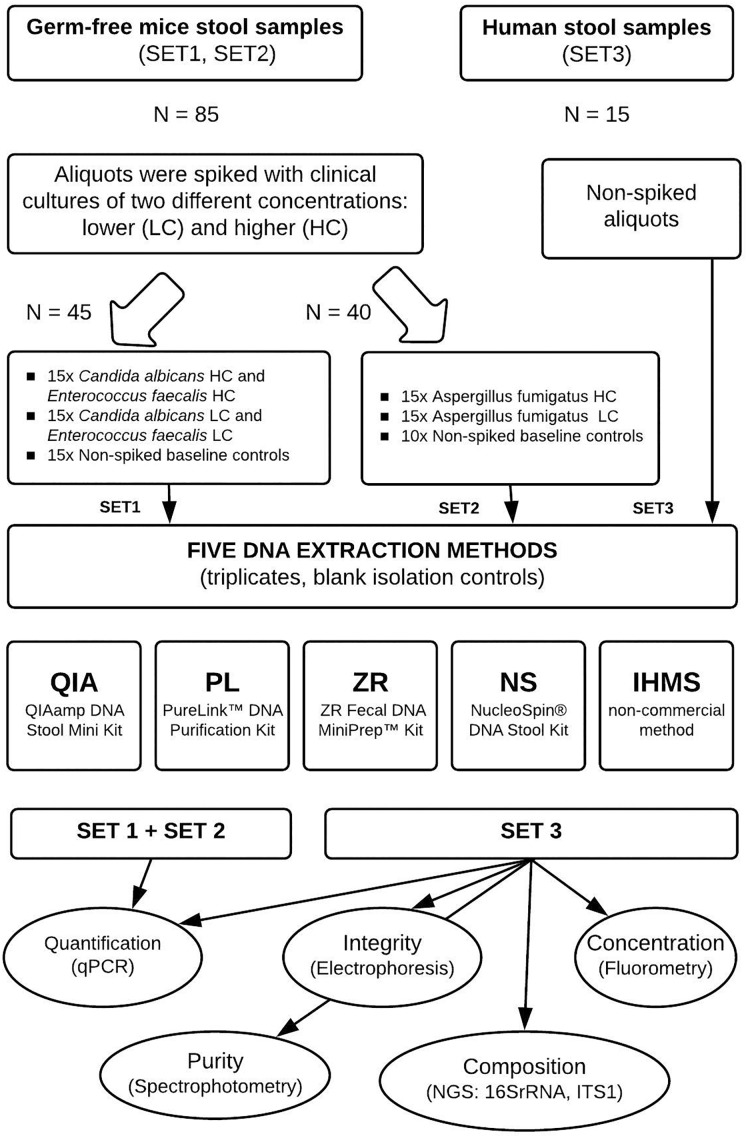
Experimental study design. The figure summarizes steps taken and the number of samples processed at each step. Two types of stool material were used for three sets of DNA extraction, differing in the spiking process. DNA was extracted from all spiked and non-spiked stool samples in triplicates (Set 2 baseline controls were processed in duplicates) using five different extraction methods. Then DNA samples were quantified using real-time PCR. DNA samples extracted from human stools were further analyzed for purity, integrity, yield, and microbial composition.

To compare simultaneous bacterial and fungal DNA extraction effectiveness, we used two types of fecal material, (i) germ-free mice feces, for their absence of viable microbiota and very low background microbial DNA load originating from diet and bedding sterilized by irradiation (Fontaine et al., 2015; Schwarzer et al., 2017), confirmed also in our experiments (Supplementary Table S1), and (ii) a single human stool stock representing a natural sample type control to ensure an identical microbial composition in all samples. Germ-free feces were inoculated with known quantities of clinical bacterial (*Enterococcus faecalis*) and fungal (*Candida albicans* and *Aspergillus fumigatus*) strains to evaluate the methods’ yields using real-time PCR. The strains were chosen as typical representatives of gram-positive, yeast and filamentous microorganisms, respectively. Human feces serving as vehicle controls were analyzed to compare DNA extraction methods in terms of integrity, purity, DNA quantity and microbial composition. Use of the single human stool sample was intended to avoid the effect of inter-individual variability observed elsewhere (Huseyin et al., 2017). The detailed study design is shown in Figure 1.

### Sample Preparation and Collection

Germ-free mice fecal samples were obtained from the Laboratory of Gnotobiology, Institute of Microbiology, Academy of Sciences of the Czech Republic. This laboratory is well-established in germ-free animal research (Kozakova et al., 2016; Schwarzer et al., 2016). A single human stool sample was donated by a healthy volunteer who signed informed consent in accordance with the Ethic Committee of the Centre for Cardiovascular Surgery and Transplantation (Protocol no. 201603).

Immediately after delivery, two grams of mice feces were homogenized using TissueRuptor^®^ (Qiagen, Germany) in 18 mL of autoclaved PBS, divided into 160 μl aliquots (*n* = 85) and frozen at -80°C until used. Then, the stool aliquots were separated into groups for two independent sets of DNA extraction. For the first set, the aliquots (*n* = 30) were simultaneously spiked with 20 μl of two different *C. albicans* concentrations (6.9 × 10^4^ or 6.9 × 10^5^ cells/ml; low or high concentration, respectively) and 20 μl of *E. faecalis* concentrations (7.7 × 10^5^ or 7.7 × 10^7^ cells/ml), resulting in a 200 μl total sample volume (Figure 1). For the second set, the aliquots (*n* = 30) were spiked with 20 μl of two different amounts of *A.* *fumigatus* (10^6^ or 10^8^ cells/ml approximately) and 20 μl of sterile water (B. Braun Medical, Inc., Germany) to preserve an equal sample volume. Stool aliquots spiked with 40 μl sterile water (*n* = 25) were included in both groups as the baseline microbial DNA load controls.

In addition, we used 200 mg of human feces, serving as a natural sample type control for the third independent DNA extraction set. The stool was homogenized in 4 mL autoclaved PBS, divided into 200 μl aliquots (*n* = 15), and frozen at -80°C until used. Dilutions were performed to make samples homogeneous and pipettable to ensure exactly the same volume (the same amount of microbiota) in all samples and thus, identical conditions for each extraction method tested.

### Methods Used for DNA Extraction

Five different fecal DNA extraction methods were evaluated in this study: QIAamp DNA Stool Mini Kit with a pre-treatment step (QIA; Qiagen, Germany), PureLink^TM^ Microbiome DNA Purification Kit (PL; Thermo Fisher Scientific, United States), ZR Fecal DNA MiniPrep^TM^ Kit (ZR; Zymo Research, United States), NucleoSpin^®^ DNA Stool Kit (NS; MACHEREY-NAGEL GmbH & Co., KG, United Kingdom) and non-commercial protocol Q, recommended by the International Human Microbiome Consortium (abbreviated as IHMS). All protocol procedures were performed according to the manufacturer’s instructions with minor differences (detailed protocols are shown in the Supplementary Material). Three independent sets of DNA extraction were performed (Figure 1), and all experimental samples were processed in triplicates for the reproducibility evaluation. Samples with sterile water were used as a negative reagent and process contamination controls. Altogether, 115 samples and reagent controls were extracted in all sets.

### Quality and Quantification of Extracted DNA

DNA concentration was determined fluorometrically on the Qubit^®^ 3.0 Fluorometer (Thermo Fisher Scientific, United States) using the Qubit^TM^ dsDNA HS Assay Kit. DNA purity was determined via 260/280 and 260/230 ratios measured on the NanoDrop 1000 (Thermo Fisher Scientific, United States). DNA integrity was determined by 0.6% agarose gel electrophoresis and visualized (Supplementary Figure S1).

### Real-Time Quantification of Bacterial and Fungal DNA

Commercial real-time PCR kit for specific *E. faecalis* detection (PrimerDesign^TM^ genesig, United Kingdom) was used to evaluate the bacterial yield using the ABI 7500 fast Real-Time PCR System (Applied Biosystems, United States).

The fungal yield was evaluated using a broad-range real-time PCR assay as described previously (Nemcova et al., 2015) using the Rotor-Gene 6000 (Corbett Research, Austria). Briefly, amplification of the ITS2 region using primers UNF1 (5′-GCATCGATGAAGAACGCAGC-3′) and UNF2 (5′-TTGATATGCTTAAGTTCAGCGG-3′) was performed with 2 × SensiFAST HRM mix (Bioline, United Kingdom) and RNase-Free Water (Qiagen, Germany).

In both real-time PCR assays, all the analyzed samples were performed in duplicates with serially diluted calibration curves. Sterile water served as no template control. Pure culture extracts served as positive controls for species verification. Altogether, 394 real-time PCR reactions for germ-free fecal samples and 60 real-time PCR reactions for human fecal samples (controls included) were performed.

Statistical analyses were conducted and visualized in R (3.4.2). One-way analysis of variance (ANOVA, function aov in R) followed by Tukey’s multiple comparison test (function Tukey HSD in R) was used to evaluate the DNA yield and real-time PCR data. *p*-values lower than 0.05 were considered significant. Log transformed data were used for fold change analysis.

### Bacterial and Fungal Composition Analysis Using NGS Sequencing

Microbial communities were profiled by *16S rDNA* and *ITS1 rDNA* amplicon sequencing using the Illumina MiSeq sequencing platform. Primers with unique barcode sequences for PCR amplification over the bacterial *16S rDNA* gene’s V3/V4 region were designed as described previously (Kubasova et al., 2017). Primer pairs ITS1F/ITS2, recommended by the Earth Microbiome Project^1^, were used with unique barcode sequences designed in this study, to amplify over the fungal internal transcribed spacer region 1 (ITS1) of the rRNA operon (Supplementary Table S2).

16S Library was constructed according to the “16S Metagenomic Sequencing Library Preparation protocol” recommended by Illumina, with minor differences. Briefly, PCR was performed with 2 × KAPA HiFi HotStart ReadyMix (Kapa Biosystems, Inc., United States) under the following conditions: initial denaturation at 95°C for 15 min, followed by 30 cycles consisting of denaturation at 95°C for 40 s, annealing at 55°C for 45 s and extension at 72°C for 60 s, with a final extension step at 72°C for 5 min. From each reaction, 5 μl were analyzed by 2% agarose gel electrophoresis. PCR products were purified using AMPure XP magnetic beads (Beckman Coulter Inc., United States) diluted into an equimolar concentration and pooled according to their unique barcode sequence, which enables multiplexing. Only samples with different barcode sequences were pooled together. Next, Illumina dual-index barcodes were added to the pooled PCR products with the Nextera XT Index Kit (Illumina, United States). Indexed PCR products were purified and pooled into the equimolar concentration prior to paired-end sequencing with MiSeq Reagent Kit v3 (600 – cycle) (Illumina), following the manufacturer’s directions.

The ITS1 Library was constructed in a similar way to the 16S Library, with the following modifications: PCR was performed with HotStarTaq Master Mix (Qiagen, Germany), 4 mM MgCl_2_ (Thermo Fisher Scientific, United States), RNase-Free Water under the following conditions: initial denaturation at 95°C for 15 min, followed by 40 cycles consisting of denaturation at 95°C for 30 s, annealing at 56°C for 45 s and extension at 72°C for 60 s, with a final extension step at 72°C for 10 min. In the first clean up step, we increased the bead amounts to 35 μl per sample to match the input volume. Then the purified PCR products were diluted to an equimolar concentration and samples with different barcode sequences were pooled together. Next, Illumina dual-index barcodes were added to the pools with the Nextera XT Index Kit (Illumina, United States). Indexed PCR pools were purified and pooled into the equimolar concentration prior to paired-end sequencing with MiSeq Reagent Kit v3 (600 – cycle) (Illumina) following the manufacturer’s directions.

### Bioinformatics and Statistical Analyses of NGS Data

For both libraries, raw sequence data analysis was carried out using QIIME (v. 1.9.1.) pipeline (Caporaso et al., 2010). 16S and ITS1 read pairs were demultiplexed based on the unique barcode sequence and then merged using the default QIIME script (join_paired_end.py). Chimeric sequences were identified using VSEARCH (v. 2.6.1) (Rognes et al., 2016) with Greengenes reference database (v. 4feb2011) for bacteria and UCHIME (v. 7.2) reference dataset for fungi. Sequences were clustered into OTUs at 97% threshold using VSEARCH 2.6.1 *de novo*. A set of sequences representing OTUs was created, and taxonomy was assigned (using script: assign_taxonomy.py) to each sequence using the Greengenes database (v. gg_13_8_otus) and Uclust (v. 1.2.22q) (Edgar, 2010) for bacteria, and using BLAST and UNITE (v. 7.2)^2^ for fungi (input sequences were searched for against a BLAST database of pre-assigned reference sequences from UNITE). This process resulted in the OTU table in BIOM format with the singletons discarded. The bacterial OTU table was filtered for OTUs, with the number of sequences less than 0.005% of the total number of sequences (Bokulich et al., 2013). The fungal OTU table was not further filtered. PyNAST (v. 1.2.2.) was used to align representative sequences to build a phylogenetic tree using FastTree (v. 2.1.3) in bacterial analysis. The non-phylogenetic metric (i.e., Bray–Curtis dissimilarity distance) was calculated for fungi due to the inapplicability of phylogenetic-based metrics (i.e., Weighted/Unweighted UniFrac distance) for ITS1 sequence analysis (Halwachs et al., 2017). Alpha- and beta-diversity calculations were performed and visualized with QIIME script core_diversity_analyses.py.

Statistical analyses were performed using online Calypso software (version 8.82) (Zakrzewski et al., 2017). Data were normalized using cumulative-sum scaling (CSS) and log2 transformation. Beta-diversity calculations were visualized using the principal coordinate analysis plots (PCoA), based on unweighted UniFrac, weighted UniFrac (bacterial data) and Bray–Curtis (fungal data) distances, and compared using the nonparametric analysis of similarities (ANOSIM) test. Moreover, the nonparametric Kruskal–Wallis and generalized linear model (GLM) tests implemented in ALDEx2 test were applied to detect differences in taxa abundances at genera level. These tests were performed with only the higher abundance taxa (>0.01% of total). The *p*-values were adjusted according to the Benjamini–Hochberg procedure. *p*-values lower than 0.05 were considered significant. To characterize method specific composition profiles, “core microbiome” measurements were performed.

## Results

Two types of fecal material were used to compare five selected DNA extraction methods. To examine method yield, DNA was extracted from germ-free mice stool samples spiked with a known amount of clinical microbial cultures and processed using specific real-time PCR.

Moreover, the human stool samples were independently analyzed for purity, integrity, yield and microbial composition to establish the most effective simultaneous bacterial and fungal DNA extraction protocol for downstream microbiome analysis (Figure 1).

### DNA Extraction From a Spiked Murine Stool Using Bacterial- and Fungal-Specific Real-Time PCR

To compare the DNA extraction methods, an artificial system was constructed. The homogenized germ-free mice stool aliquots were spiked with verified clinical bacterial and/or fungal cultures, each in two defined concentrations differing in one to two orders of magnitude. Non-spiked stool aliquots were used as a baseline microbial load, and sterile water was used as a blank control. All extractions were performed in triplicates and compared using bacterial- and fungal-specific real-time PCR, resulting in 394 reactions.

**FIGURE 2:**
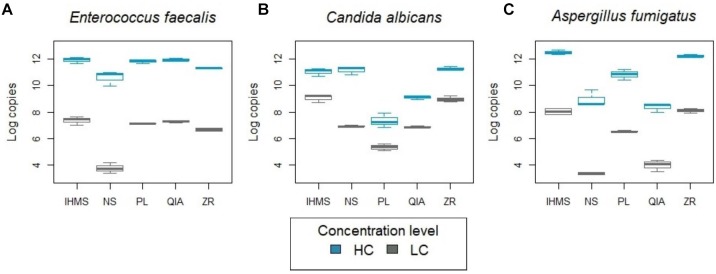
Comparing extracted DNA yield using real-time PCR. *X*-axes represent extraction method types. *Y*-axes represent DNA yield determined by real-time PCR. Mice stool samples were spiked with clinical cultures of two different concentrations – lower (LC; gray) and higher (HC; blue): **(A)** *E. faecalis* (10^5^ and 10^7^ cells/ml), **(B)** *C. albicans* (10^4^ and 10^5^ cells/ml) and **(C)** *A. fumigatus* (10^6^ and 10^8^ cells/ml). Note that input concentration volumes differ in one order of magnitude in *C. albicans* and two orders of magnitude in *E. faecalis* and *A. fumigatus* assays.

All methods varied in terms of technical reproducibility, but the variability among replicates was considerably lower than among the same samples extracted by different methods. Extraction from the low-concentrated (LC) and high-concentrated (HC) communities showed similar patterns (Figure 2). However, microbial DNA recovery was significantly influenced by the DNA extraction method.

A real-time PCR analysis of the extracted bacterial DNA revealed a similar amount of extracted DNA in IHMS, PL, QIA and ZR methods, but a significantly lower DNA yield for the NS method (LC and HC level; *p* < 0.001 and *p* < 0.05, respectively) (Figure 2A). Moreover, real-time PCR analysis indicated moderate PCR inhibition by extraction with the ZR method (Supplementary Figure S2).

Extracting fungal DNA yielded variable results between the methods used (Figure 2B,C) and between *C. albicans* and *A. fumigatus*, selected as typical yeast and mold representatives. For both species, the IHMS and ZR methods provided the highest DNA yields consistently (Figure 2B,C). The significantly lower yields (*p* < 0.001) were produced by QIA and PL methods in both concentration levels. The QIA method produced higher DNA yields for *C. albicans* (Figure 2B) and the PL method was more successful in *A. fumigatus* extraction (Figure 2C). The NS method results were inconsistent and were species and load-dependent (Figure 2B,C). It is also notable to mention that the ZR method’s profile was not observed as being inhibited in any fungal assays.

During *E. faecalis* DNA detection, a positive signal was occasionally captured in one of the baseline controls’ real-time PCR duplicates (once in both, *n* = 5/15) across methods, with values lower than 1 copy (Supplementary Table S1). Therefore, it was considered as being close to the detection limit, and thus negligible. No *E. faecalis* DNA signal was captured in blank controls. During fungal DNA detection, a positive signal was captured in all baseline controls (*n* = 25). However, only the ZR method significantly differed (*p* < 0.05) from every other method, resulting in a higher fungal DNA yield. In addition, fungal DNA was also detected in the blank controls (*n* = 8/10) with values lower than 10 copies for four methods (QIA, PL, NS, and IHMS) and 100 copies for the ZR method (Supplementary Table S1).

### Analyzing of Quantity, Purity and Integrity of Extracted Total DNA From Human Stool Samples

Using all five extraction methods, genomic DNA was successfully isolated from the human feces serving as natural sample type controls. While the performed technical triplicates were reproducible, the DNA yields and quality of DNA extracted differed between methods (Table 1). The highest DNA yields were obtained using the IHMS and ZR methods, while NS, QIA and PL methods resulted in five times lower amounts (*p* < 0.05). In addition, DNA extracted with the IHMS, PL, or NS methods were not contaminated with RNA, protein, or organic contents, while ZR extraction showed the lowest DNA purity (Table 1).

**Table 1:** Characteristics of total DNA from human stool samples using various extraction methods.

	DNA concentration	DNA purity		DNA integrity
Method	ng/μl	A_260/280_	A_260/230_	Band intensity
PL	6.52 ± 0.78	1.72 ± 0.23	0.84 ± 0.17	+
NS	8.63 ± 0.32	1.78 ± 0.05	1.35 ± 0.16	+
QIA	8.17 ± 1.10	2.20 ± 0.10	0.51 ± 0.05	±
ZR	36.05 ± 1.43	1.38 ± 0.02	0.58 ± 0.06	+
IHMS	28.60 ± 2.94	2.06 ± 0.07	1.77 ± 0.06	++


*DNA concentration and DNA purity mean and standard deviation. Band intensity (as visualized by electrophoresis Supplementary Figure S1) of DNA integrity. PL: PureLink^TM^ Microbiome DNA Purification Kit; NS: NucleoSpin^®^ DNA Stool Kit; QIA: QIAamp DNA Stool Mini Kit; ZR: ZR Fecal DNA MiniPrep^TM^ Kit; IHMS: protocol Q. ±, very faint band; +, faint band; ++, normal-intensity band.*

Next, to verify fungal DNA presence in the human stool DNA extracts, fungal-specific real-time PCR analysis was performed (Supplementary Figure S3). All extractions were performed in triplicates, in the same way as fungal assays in the murine system. For human feces, a similar “yield pattern” was observed to the assay with a high *C. albicans* community concentration, which indicates a yeast dominance in the examined human stool samples. Blank controls were also quantified, resulting in mean values 3.3 logs lower than the samples (Supplementary Table S1).

In general, all evaluated DNA extraction methods produced a sufficient DNA quantity and quality for subsequent human fecal sample NGS analysis.

### NGS Analysis of Bacterial Diversity and Composition From Various DNA Extractions of Human Stool

The pooled 15 samples (5 method; 3 replicates) returned a total of 384579 *16S rDNA* gene sequence reads after raw sequence filtration (see section “Materials and Methods”) and chimera removal. These reads (range 71124–83383 per method; range 20938–30270 per replicate) were distributed into 60 bacterial taxa assigned at genus level (Supplementary Table S3). Only 21 unassigned reads (0.005% of the total) passed filter criteria. Forty-nine taxa were detected in all extracted samples, and thus represented a “core microbiome.” Among the other eleven taxa, no unique method-associated taxon was observed (Supplementary Table S3). The blank controls, also sequenced, contained a very low number (*n* = 1018) of sequencing reads. However, *Acinetobacter* reads (*n* = 943) detected in the ZR method’s blank control, were also reflected as increased relative abundance of *Acinetobacter* in the ZR method’s microbial composition profile (1.2% in ZR vs. 0.003% in others). The complete taxa list detected in the blank controls is shown in Supplementary Table S4.

**FIGURE 3:**
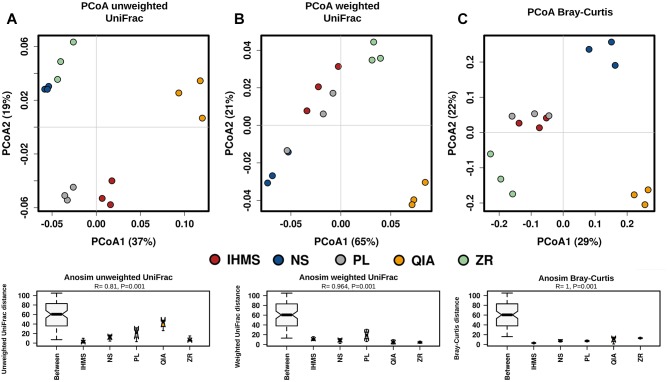
Comparison of beta-diversity between DNA extraction methods. Principal coordinate analysis (PCoA) plot based on the unweighted **(A)**, and weighted **(B)** UniFrac distance for bacteria; PCoA plot based on Bray–Curtis dissimilarity **(C)** for fungi. Each PCoA plot is accompanied by an analysis of similarity (ANOSIM) of five methods using appropriate distance matrix. The R-values > 0 that show the significant differences in the between-methods communities compared those in the within-methods communities. The *p*-values < 0.05 indicate a significantly different level between methods. *Y*-axes represent appropriate dissimilarity matrix values.

Bacterial alpha-diversity was similar between the IHMS, NS, PL, and ZR methods, while the QIA method showed the lowest rarefaction curve (Supplementary Figure S4A). Principal coordinate analysis (PcoA) of beta-diversity revealed observable clusters according to the method, using both the unweighted (Figure 3A) and weighted (Figure 3B) UniFrac distance. Moreover, analyses of similarity (ANOSIM), performed for both UniFrac distance matrices, confirmed significant variability among the used extractions (*p* < 0.001; Figure 3A,B).

**FIGURE 4:**
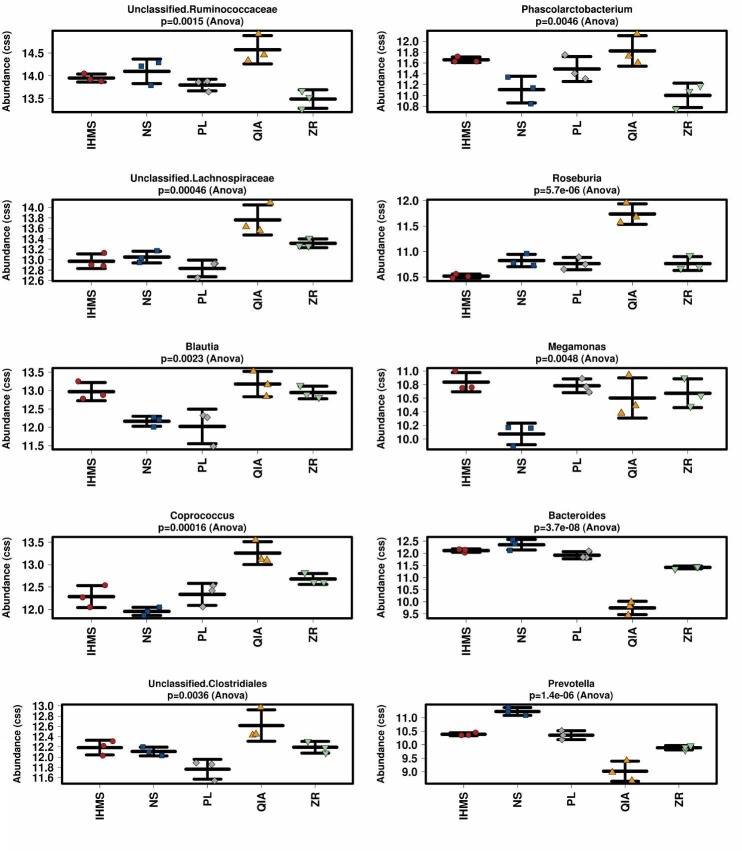
Stripchart comparison of cumulative-sum scaling (CSS) and log2 transformation data from bacterial taxa according to extraction method. Figure shows ten most abundant bacterial taxa significantly different between methods. Error bars in stripcharts visualize standard deviation.

To compare taxa abundance differences at genus level between methods, we performed Kruskal–Wallis and GLM tests with adjusted *p*-values according to the Benjamini–Hochberg procedure. We found that the examined taxa (*n* = 52; relative abundance > 0.01% of total) varied greatly between extraction methods. We detected significant differences (*p* < 0.05) in 19/52 taxa applying the Kruskal–Wallis test and 42/52 taxa applying the GLM test (Supplementary Table S5). The ten most abundant bacterial taxa significantly differing between methods are shown in Figure 4.

### NGS Analysis of Fungal Diversity and Composition From Various DNA Extractions of Human Stool

The same 15 pooled samples (5 method; 3 replicates) were analyzed for their ITS1 target. Chimeric and singleton sequence removal resulted in a total 483224 sequence reads. These reads (range 73630–119305 per method; range 23218–44101 per replicate) were distributed into 72 fungal taxa assigned at genus level. Only 0.06% of the total reads (*n* = 294) were not assigned. Two taxa (i.e., unidentified *Dipodascaceae* and *Helotiales*) with an average relative abundance of 99% were dominant in all extracted samples and the other 70 taxa, (altogether <1% of total abundance) varied between methods. Among them, a majority (*n* = 47) of the taxa were consistent between replicates (Supplementary Figure S5), while the remaining 23 taxa were occasionally detected in one of the methods’ replicates and were considered environmental/kit contamination. Moreover, most of these rare taxa (36/72) were also present in the blank controls (Supplementary Table S4), although positive signals detected by real-time PCR were several logs beyond the analyzed samples’ signals (Supplementary Table S1).

Contrary to bacteria, the fungal “core microbiome” constituted of only five taxa (Supplementary Table S3) and the most of the recovered taxa (*n* = 27; out of 47) were uniquely detected by only one method. This variable fraction was probably responsible for the observed alpha-diversity differences (Supplementary Figure S4B).

Fungal beta-diversity analysis showed similar results to the bacterial samples (Figure 3). PCoA based on non-phylogenetic Bray–Curtis dissimilarity revealed distinct clusters according to each method (Figure 3C), and subsequent ANOSIM analysis confirmed lower variability within methods than between them (Figure 3C).

Similar to bacteria, we performed Kruskal–Wallis and GLM tests with adjusted *p*-values according to the Benjamini–Hochberg procedure to assess the differences between each method’s taxa abundances. Only taxa at genus level with a higher relative abundance than 0.01% of total (*n* = 16) were tested. We detected significant differences (*p* < 0.05) in 15/16 taxa by applying a GLM test, although there were no significant differences after applying a Kruskal–Wallis test (Supplementary Table S5).

## Discussion

The purpose of this study was to compare the efficiency of five different DNA extraction methods widely used in human gut microbiome research, with a particular focus on simultaneous bacterial and fungal analysis, and to address the impact of laboratory/reagent contamination on the microbial profile. Overall, all methods generated a sufficient DNA yield and quality for PCR amplification of both the bacterial and fungal target regions. Thus, the absence of a fungal PCR product due to an inefficient extraction protocol, as described previously (Huseyin et al., 2017), was not an issue in this study. Despite the fact that the methods varied in producing genomic DNA yields, they had no obvious effect on bacterial or fungal alpha-diversity, which is in line with the findings of others (Knudsen et al., 2016; Huseyin et al., 2017; Rintala et al., 2017; Lim et al., 2018).

In view of the fact that the fecal genomic DNA is not exclusively microbial, but also originates from the host and food, we performed species-specific assays. We used artificial system, germ-free mice fecal samples spiked with several species to evaluate the DNA extraction method’s impact on microbial recovery. The gut colonizer *E. faecalis* was selected due to its gram-positive cell wall, which is generally harder to lyse, as discussed earlier (Maukonen et al., 2012; Santiago et al., 2014). Surprisingly, we did not reveal any major impact from the different methods’ performance on *E. faecalis* DNA recovery, even though IHMS, PL and QIA methods generated a better outcome, owing to a significantly lower yield and inhibited PCR when using NS and ZR methods, respectively (Figure 2A and Supplementary Figure S2). Yield differences could be explained by neither mechanical, nor enzymatic lysis selection, since all protocols except QIA, incorporated a bead-beating step. However, the absence of a bead-beating step or InhibitEX tablet use in the QIA protocol was reflected in a lower bacterial diversity estimation (Supplementary Figure S4A), also documented in Costea et al. (2017).

Contrary to bacteria, fungal cell walls are more complex with manifold sensitivity to lysis (Fredricks et al., 2005). In addition, different fungal groups produce different type of cells like hyphae, yeast cells, and spores. Therefore, finding one method capable of sufficiently extracting DNA from all fungal types is challenging. Thus, to examine the methods’ fungal lysis capability, *C. albicans* and *A. fumigatus* were selected as representatives of the two major fungal groups, yeast and filamentous fungi for fungal assays. Here, we detected a great variation between the methods’ performance. In addition, DNA extraction efficiency was not only method- but also species-dependent, which is consistent with Rittenour et al. (2012) observation, comparing DNA extraction kits for environmental dust samples. Our data also supported the results of another study (Fredricks et al., 2005), where DNA extraction was less effective from *A. fumigatus* than *C. albicans*. Here, *A. fumigatus* yield was approximately one order of magnitude lower for all methods (Figure 2). Subsequent human fecal samples analysis revealed a similar “yield pattern” to the *C. albicans* assay, and we thereby independently validated the methods’ performance in yeast assays since the most dominant taxon in human feces was the *Dipodascaceae* yeast family.

We realize the fact that the results might be biased by the small number of microbial strains used as a limitation of our study. However, our experimental murine system setup was designed to allow the exploration of the relative differences between each method’s performance and reproducibility. Since one of the presumptions of successful DNA extraction is microbial cell wall disruption, we selected three microbial strains to represent three major groups differing in cell wall thickness and composition. Germ-free feces were then spiked with two of these strains’ concentrations to explore each method’s performance in low and high concentrations. All spiked samples were extracted in triplicates to ensure method reproducibility. Non-spiked samples were analyzed to establish the samples’ baseline DNA loads. No template controls were processed to control contaminations during the extraction process. In fact, we were focused on the stability and efficiency of the methods’ performance rather than multiple strain detection in a less thorough methodical setting. In addition, human feces serving as a vehicle control were analyzed to provide a view of the methods’ performance under real conditions.

All in all, the best performance in the bacterial and fungal DNA yield recovery context was observed when employing the IHMS method. Admittedly, the highest yield does not necessarily mean the most accurate results in terms of microbial composition. However, since fungi form a marginal community in fecal samples, the capability of recovering fungal DNA seems to be an advantage in fecal mycobiome research.

As has been previously reported (Wesolowska-Andersen et al., 2014; Costea et al., 2017), selecting the extraction method significantly impacted bacterial composition. The major differences were observed in taxa relative abundance rather than particular taxa detection, and thus no unique method profile was uncovered. In addition, it seems that PL and IHMS composition profiles were the most similar to each other, as shown in Figure 3 and 4, suggesting the possible results are comparable when employing these two methods in bacterial microbiome research.

Contrary to bacterial analysis, interpreting the fungal results was more challenging. As two previous studies suggested, the extraction protocol might not be critical in fungal composition assessment (Huseyin et al., 2017; Angebault et al., 2018), however, a low number of protocols were tested in these studies. In our study, unfortunately, the fungal communities were dominantly (>99%) constituted by only two taxa (i.e., unidentified *Dipodascaceae* and *Helotiales*), which represents a limitation in fungal diversity analyses, as only 1% influenced the outcome. Both taxa were previously detected in human stool samples (Hallen-Adams and Suhr, 2016; El Mouzan et al., 2017; Mandarano et al., 2018). The *Dipodascaceae* yeast family is capable of colonizing the human gut, contrary to the *Helotiales* order, which harbors many plant endophytes (Hynson and Bruns, 2009), and thus probably represented a food contaminating DNA, rather than viable gut colonizers. Although the examined methods did not significantly differ in the DNA recovery of these two taxa, it would be preliminary to generally claim that selecting the extraction method has no impact on mycobiome outcome, regarding the results from fungal quantitative assays (Figure 2 and Supplementary Figure S3). In addition, unique method profiles were observed when analyzing the remaining fraction (Figure 4 and Supplementary Figure S5). We consider these rare communities to be kit/reagent contaminations, since the majority were also detected in the blank controls. Moreover, these contaminations artificially increased alpha-diversity estimates, as shown in Supplementary Figure S4, where DNA extracted using the IHMS and NS methods was contaminated by the least taxa.

We also assume that due to the methodological differences between bacterial and fungal analysis (i.e., higher number of PCR cycles required), the presence of the reagents’ fungal contamination may be more common in mycobiome datasets than bacterial ones. It has been previously discussed that the bacterial contamination of kits/laboratory reagents may be an issue when analyzing a sample with low biomass (Salter et al., 2014), contrary to gut microbiome analysis where high bacterial baseline concentrations in fecal samples are less prone to it (Kim et al., 2017). Given the low fungal biomass present in some fecal samples, interpreting data may suffer an analogous bias. Thus, the appropriate blank controls should be included and processed in all mycobiome analyses to distinguish between real sample and contamination profiles. Sample fungal load quantification by real-time PCR might also be helpful to predict potentially contamination-prone samples. At the same time, it is necessary to say that incorporating an appropriate sequence filtration step would significantly help to decrease the numbers of contamination taxa.

## Conclusion

Our understanding of the human gut microbiome role in health and disease depends on obtaining reliable and comparable microbial data of both bacterial and fungal communities. To achieve this goal, using an appropriate methodology is an important step. Previous efforts in standardizing methodology for bacterial microbiome research lead to the recommendation of using the IHMS protocol Q worldwide, to ensure data reproducibility and comparability. In this study, we evaluated and compared the impact of five different DNA extraction methods including the IHMS protocol Q, on the representation of fecal bacterial and fungal communities, with an emphasis on applying the method for use on both communities. In our experimental design, the evaluated DNA extraction methods significantly differed in the efficiency and quality of the isolated DNA, which affected the identified stool microbiome composition. We also discussed the impact of fungal contaminations revealed in kit reagents. Overall, based on the data obtained, we suggest using the DNA extraction protocol “IHMS protocol Q,” which is suitable for simultaneously analyzing both the bacterial and fungal gut community.

## Ethics Statement

This study was carried out in accordance with the recommendations of Committee of the Centre for Cardiovascular Surgery and Transplantation (Protocol no. 201603), with written informed consent from all subjects. All subjects gave written informed consent in accordance with the Declaration of Helsinki. The protocol was approved by Committee of the Centre for Cardiovascular Surgery and Transplantation.

## Author Contributions

KF and TF conceived the initial project design with inputs from EN and MC. KF performed all experiments and statistical analyses of the data with significant contributions from EN and HG. MR performed the bioinformatics analysis of the data. ML provided bioinformatics assistance. KF, HG, and TF wrote the manuscript. JB and DS significantly contributed to the final preparation of the manuscript. All authors revised and approved the final manuscript.

## Conflict of Interest Statement

The authors declare that the research was conducted in the absence of any commercial or financial relationships that could be construed as a potential conflict of interest.

## Footnotes

1 http://www.earthmicrobiome.org/protocols-and-standards/its/

2 PlutoF biodiversity platform Available at: https://plutof.ut.ee/#/datacite/10.15156%2FBIO%2F587476
