# Supplementary material for: The Impact of DNA Extraction Methods on Stool Bacterial and Fungal Microbiota Community Recovery
Source: Front Microbiol. 2019 Apr 17;10:821. doi: 10.3389/fmicb.2019.00821 (PMC6479168; doi:10.3389/fmicb.2019.00821)
Supplement: Supplementary file 1 [file Table_1.DOCX]

Supplementary Material

The impact of DNA extraction methods on stool bacterial and fungal microbiota community recovery

**Kristýna Fiedorová^1, 2, 5^, Matěj Radvanský^3^, Eva Němcová^1^, Hana Grombiříková^1^, Juraj Bosák^4^, Michaela Černochová^1^, Matěj Lexa^3^, David Šmajs^4^, Tomáš Freiberger^1, 2, 5*^**

*** Correspondence:** Tomas Freiberger: tomas.freiberger@cktch.cz

# List of Supplementary Material

1. Supplementary Methods: A detailed description of protocol procedures
2. Supplementary Figures:

- Figure S1: Integrity of total DNA
- Figure S2: Real-time PCR plot of *E. faecalis* detection
- Figure S3: Comparison of fungal DNA yield from human stool
- Figure S4: Alpha-diversity
- Figure S5: Comparison of fungal relative taxa abundance

1. Supplementary Tables:

- Table S1: Real-time PCR data
- Table S2: Sequences of fungal primers
- Table S3: Core microbiome analyses
- Table S4: Blank controls
- Table S5: Taxa comparison

# Supplementary Methods

## Extractions of microbial DNA from fecal samples were performed using following protocols:

1**.** QIAamp DNA Stool Mini Kit (QIA; Qiagen, Germany)

2. PureLink™ Microbiome DNA Purification Kit (PL; Thermo Fisher Scientific, USA)

3. ZR Fecal DNA MiniPrep™ Kit (ZR; Zymo Research, USA)

4. NucleoSpin® DNA Stool Kit (NS; MACHEREY-NAGEL GmbH & Co. KG, UK)

5. Non-commercial IHMS protocol Q

### QIA:

The following reagents were added to 200 µL of diluted feces and held for 30 min at 37°C prior DNA extraction:

• 20 µL of Lysozyme (180 mg/mL) – Roche

• 20 µL of Lyticase (5U/µL) - Sigma-Aldrich

• 20 µL of Lysostaphin (1.8 mg/mL) - Sigma-Aldrich

• 130 µL of buffer lysis (PCR-grade H2O; 500 mM Na2EDTA; 1M Tris; Triton X-100) – Sigma-Aldrich

Buffer ASL (1.4 mL) was added to the suspension, homogenized and heated the for 5 min at 95 ˚C. The suspension was vortexed for 15 s and centrifuged at 14,000 x g for 1 min. Supernatant (1.2 mL) was transferred into a new tube along with an InhibitEX Tablet, homogenized until completely dissolved and incubated at room temperature for 1 min. The tube was centrifuged for 3 min, all supernatant was transferred into a new tube and centrifugation was repeated. Supernatant (200 μL) and Buffer AL (200 μL) were added into a new 1.5 mL tube with 15 μL Proteinase K followed by vortexing for 15 s and incubation at 70 ˚C for 10 min. Ethanol (96-100%; 200 μL) was added to the lysate and vortexed. The lysate was applied to the QIAamp spin column and centrifuged for 1 min, collection tube containing the filtrate was discarded. The column was washed with 500 μL of Buffer AW1 and subsequently by 500 μL of Buffer AW2, the last centrifugation step was repeated with an empty collection tube. AE Buffer (100 μL) was applied to the column and incubated for 1 min at room temperature, then centrifuged for 1 min and DNA was eluted into a new 1.5 mL tube. DNA was stored at -20 ˚C until processing.

### PL:

Diluted feces (200 µL) were added, along with 600 µL of S1–Lysis Buffer to the Bead Tube and vortexed. S2–Lysis Enhancer (100 µL) was added to the sample, briefly vortexed and incubated at 65°C for 10 min. The suspension was homogenized by bead beating for 10 minutes at maximum speed on the Vortex-Genie 2 mixer (MO BIO Laboratories, Inc., USA), then centrifuged at 14,000 x g for 5 min. Supernatant (400 µL) was transferred into a new tube and vortexed along with 250 µL of S3–Cleanup Buffer, then centrifuged for 2 min. Supernatant (500 µL) was transferred into a new tube and briefly vortexed along with 900 µL of S4–Binding buffer. Lysate was applied to the spin column-tube and centrifuged for 1 min. The filtrate was discarded, and the step was repeated with the remaining lysate. The column was washed with 500 µL of S5–Wash Buffer and centrifuged for 1 min. Centrifugation step was repeated with an empty collection tube. S6-Elution Buffer (100 µL) was applied to the column and incubated at room temperature for 1 min, then centrifuged for 1 min and DNA was eluted into a new tube. DNA was stored at -20 ˚C until processing.

### ZR:

Diluted feces (200 µL) were transferred into a ZR BashingBeadTM Lysis Tube along with 750 µL of Lysis Solution. The suspension was homogenized by bead beating for 10 min at maximum speed on the Vortex-Genie 2 mixer (MO BIO Laboratories, Inc., USA), then centrifuged at 10,000 x g for 1 min. Supernatant (400 µL) was applied to the Zymo-SpinTM IV Spin Filter and centrifuged at 7,000 x g for 1 min. Fecal DNA Binding Buffer (1.2 mL) was added to the filtrate in the Collection Tube. Later, 800 µL of the mixture was applied to the Zymo-SpinTM IIC Column and centrifuged at 10,000 x g for 1 min. This step was repeated after flow-through discarding. DNA Pre-Wash Buffer (200 µL) was applied to the Zymo-SpinTM IIC Column and centrifuged for 1 min. Later the column was washed with 500 µL Fecal DNA Wash Buffer and centrifuged for 1 min. Next, 100 µL of DNA Elution Buffer was applied to the column and incubated for 2 min, then centrifuged at 10,000 x g for 30 s to elute DNA. Eluted DNA was applied to the Zymo-SpinTM IV-HRC Spin Filter, centrifuged at 8,000 x g for 1 min and stored at -20 ˚C until processing.

### NS:

Diluted feces (200 µL) were transferred into a NucleoSpin ® Bead Tube Type A along with 850 µL of ST1 Buffer and shaked horizontally 2-3 s, then incubated at 70 °C for 5 min. The tube was vortexed for 10 min at maximum speed on the Vortex-Genie 2 mixer (MO BIO Laboratories, Inc., USA), then centrifuged at 13,000 x g for 3 min. The supernatant (600 µL) was transferred into a new tube along with 100 µL of ST2 Buffer, then vortexed for 5 s, incubated for 5 min at 4 °C and centrifuged at 13,000 x g for 3 min. Lysate (550 µL) was applied to the NucleoSpin ® Inhibitor Removal Column, then centrifuged at 13,000 x g for 1 min. ST3 Buffer (200 µL) was added into the column and vortexed for 5 s. The column was placed in a new collection tube along with 700 µL sample, then centrifuged at 13,000 for 1 min. The washing of the column was performed using several wash buffers (STD3, STD4 and STD5) and centrifugations steps (13,000 x g for 1 min) after each round, then last centrifugation step was repeated with an empty collection tube at 13,000 x g for 2 min. Next, 100 µL of SE Buffer was applied to the column and centrifuged at 13,000 x g for 1 min to elute DNA. DNA was stored at -20 ˚C until processing.

### IHMS:

Diluted feces (200 µL) were transferred into a 2 mL tube containing 0.6 g of sterile zirconia beads (BioSpec, Inc., USA) - 0.1 and 0.5 mm dia along with 1 mL ASL lysis buffer (QIA chemistry). The solution was incubated at 95 °C for 15 min and vortexed for 10 min at maximum speed on the Vortex-Genie 2 mixer (MO BIO Laboratories, Inc., USA), then centrifuged at 16,000 x g for 5 min at 4 °C. Supernatant was transferred into a new 2 mL tube. The pellet was mixed with 300 µL ASL lysis buffer and the previous steps were repeated. Supernatants were pooled and along with 260 µL of 10M ammonium acetate, vortexed and incubated on ice for 5 min, then centrifuged at 16,000 x g for 10 min at 4 °C. The supernatant was divided into two 1.5 mL tubes containing 750 µL of isopropanol, vortexed, incubated on ice for 30 min and centrifuged at 16,000 x g for 15 min. The supernatant was removed and pellet was washed with 70% EtOH (0.5 mL) and dried under vacuum. Next, the pellets were dissolved in 100 µL of TE (Tris-EDTA) buffer and aliquots were pooled. In the following steps, QIA chemistry and protocol was used. Solution (200 μL) and Buffer AL (200 μL) were added into a new 1.5 mL tube containing 15 μL Proteinase K followed by vortexing for 15 s and incubation at 70 ˚C for 10 min. Ethanol (96-100%; 200 μL) was added to the lysate and vortexed. Lysate was applied to the QIAamp spin column and centrifuged for 1 min, collection tube containing the filtrate was discarded. The column was wash with 500 μL of Buffer AW1 and subsequently by 500 μL of Buffer AW2, last centrifugation step was repeated with an empty collection tube. AE Buffer (100 μL) was applied to the column and incubated for 1 min at room temperature, then centrifuged for 1 min and DNA was eluted into a new 1.5 mL tube. DNA was stored at -20 ˚C until processing.

# Supplementary Figures

**Supplementary Figure 1.** Integrity of total DNA extracted from human stool replicates using various extraction methods. Band intensity visualized by gel electrophoresis. BC: Blank controls.

**
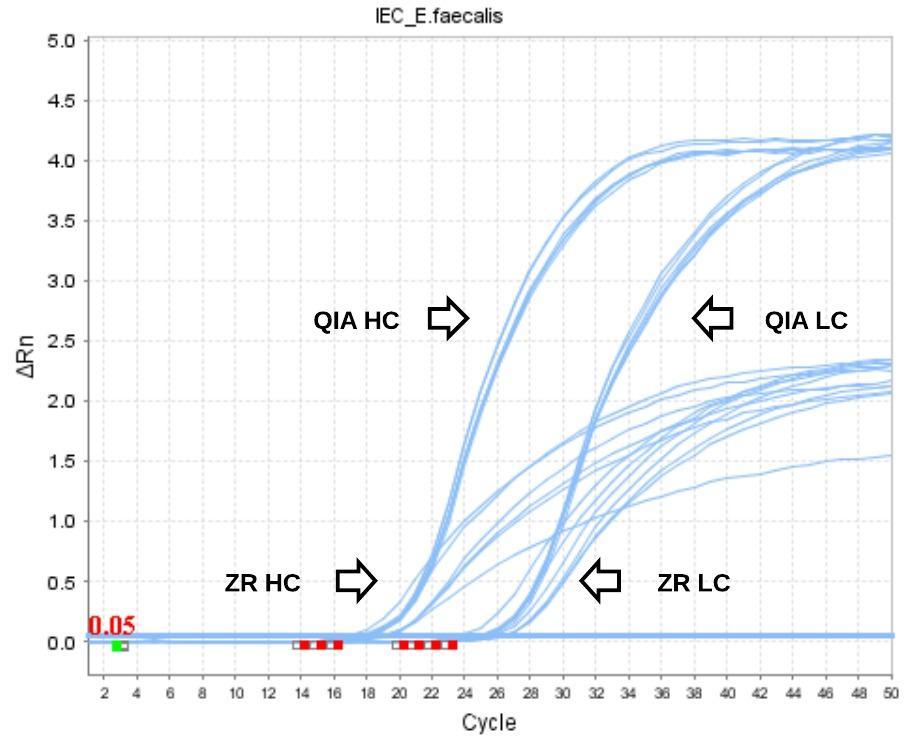
**

**Supplementary Figure 2.** Real-time PCR plot of *E. faecalis* detection. Two methods (QIA, ZR) are visualized in two different concentration levels (LC, HC). ZR reached lower maximum during real-time PCR amplification and revealed different curve shape, indicating PCR inhibition.

**
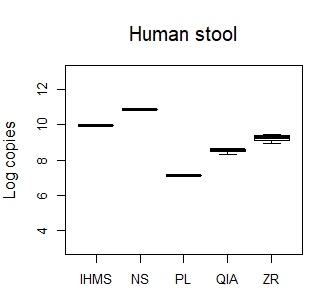
**

**Supplementary Figure 3.** Comparison of fungal DNA yield using real-time PCR from human stool sample. X-axis represents extraction method types. Y-axis represents DNA yield determined by real-time PCR.

**
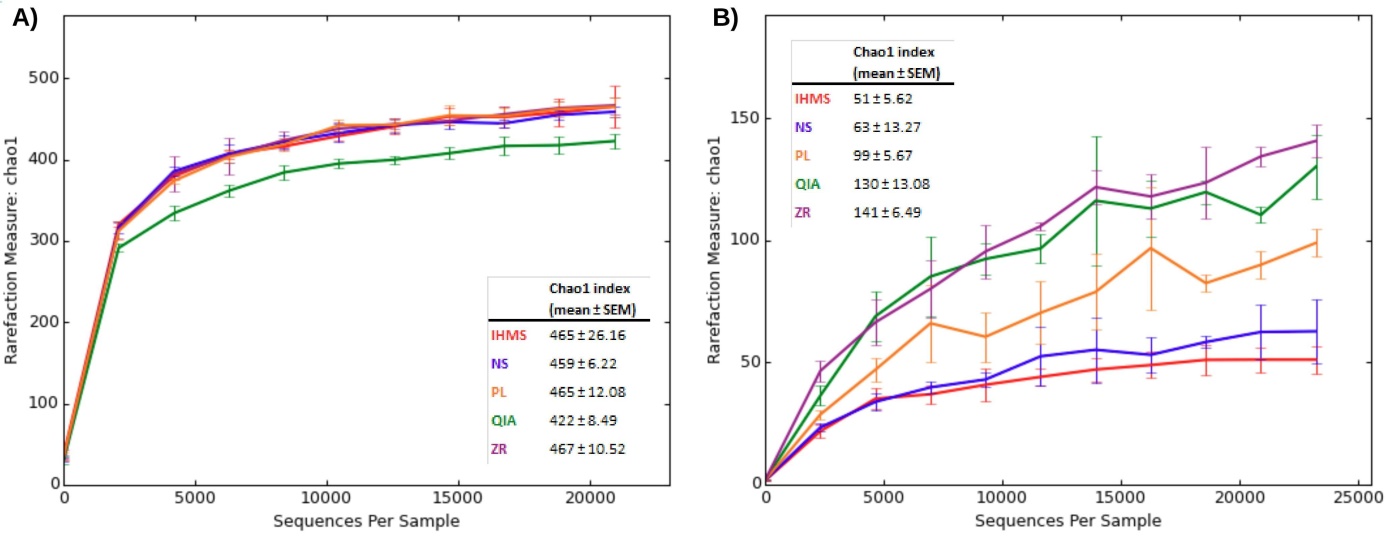
**

**Supplementary Figure 4.** Comparison of alpha-diversity between DNA extraction methods. Rarefaction curve for Chao1 index against sequences per sample calculated for bacteria **(A)** and fungi **(B)**. Note, no additional sequence filtration step was implemented in the fungal analysis.


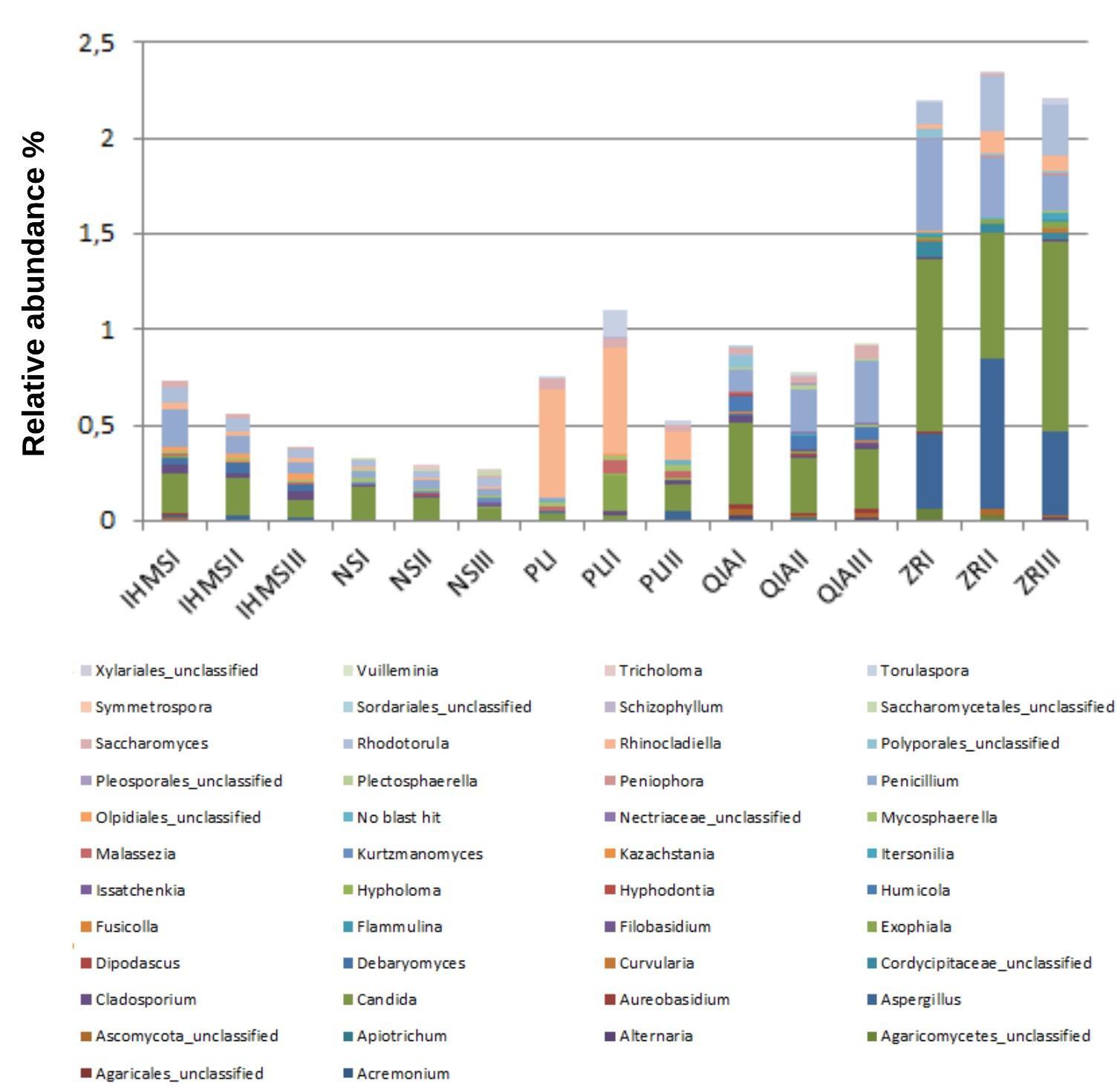


**Supplementary Figure 5.** Comparison of the fungal relative taxa abundance between DNA extraction methods. Fungal sequences were dominantly (>97.5%) constituted by two taxa (unidentified *Dipodascaceae* and *Helotiales*). Barplot represents remaining fraction (n=46; <2.5%) present in at least two replicates for one method.
